# A New *Trioza* Species (Hemiptera: Triozidae) from Japan Associated with *Urtica* (Urticaceae) †

**DOI:** 10.3390/insects17060606

**Published:** 2026-06-09

**Authors:** Hiromitsu Inoue

**Affiliations:** Institute for Plant Protection, National Agriculture and Food Research Organization, Akitsu, Higashihiroshima 739-2494, Hiroshima, Japan; inoue.hiromitsu603@naro.go.jp

**Keywords:** COI, DNA barcoding, jumping plant-lice, new species, phylogeny, psyllid, Psylloidea, Sternorrhyncha, taxonomy

## Abstract

A nettle-feeding psyllid of the genus *Trioza* (Hemiptera: Triozidae) was discovered in Hokkaido, northern Japan. Although the European species *Trioza urticae* has been reported from Japan in several databases and publications, these records are based on erroneous literature citations rather than examined specimens. Adult and immature materials collected from *Urtica platyphylla* were examined morphologically and analysed using mitochondrial cytochrome *c* oxidase subunit I (COI) DNA barcoding. The Japanese species is closely related to Palaearctic *T. urticae* and Nearctic *T. albifrons* but is consistently distinguishable by several adult and immature morphological characters. In addition, COI sequences show large interspecific divergence (≥12%) among the three species, supporting its distinct taxonomic status. The Japanese species is therefore described as *Trioza miyatakei* sp. nov., and Japan is formally excluded from the distribution range of *T. urticae*. These results indicate that records of *T. urticae* in East Asia surrounding Japan require re-examination using both morphology and DNA barcoding. A diagnostic key to all known *Urtica*-feeding species of *Trioza* worldwide is provided. Furthermore, the Chinese species *Triozopsis huai* Li, 2011 is transferred to *Trioza* as *Trioza huai* (Li, 2011) comb. nov.

## Simple Summary

Psyllids, or jumping plant-lice, are tiny insects that live on specific host plants and are often difficult to distinguish because many species are morphologically similar. In the present study, a new psyllid species, *Trioza miyatakei*, living on a native nettle plant in northern Japan is identified and described. Previous publications have listed a well-known European species as occurring in Japan; however, these records resulted from erroneous citations of earlier literature rather than the misidentification of Japanese specimens. This study aims to identify the nettle-feeding psyllid species in Japan by carefully comparing its morphological features and genetic information with those of closely related species in Europe and North America. Although subtle, stable morphological differences and, in comparison, significantly greater DNA differences are found, indicating that the Japanese population represents a distinct species new to science. Therefore, Japan should be excluded from the distribution range of well-known European species. Accurate species identification is essential for understanding biodiversity, ensuring reliable distribution records, and providing a sound basis for future ecological and plant protection studies. This study demonstrates the importance of combining morphological observations with genetic analyses to better understand the natural world.

## 1. Introduction

Jumping plant-lice, or psyllids, are small phloem-feeding hemipterans belonging to the superfamily Psylloidea, with over 4200 described species worldwide [1]. They are highly host-specific, with most species being monophagous (feeding on only a single plant species) or oligophagous (feeding on only a few closely related plant species within a single genus or family). Furthermore, related psyllids often utilise related plant species as hosts [2].

Psyllids associated with nettles, *Urtica* (Rosales: Urticaceae), belong exclusively to the family Triozidae, with seven species recognised worldwide [3,4,5]; however, only two species—*Trioza urticae* (Linné, 1758) and *Trioza albifrons* Crawford, 1910—have been confirmed as developing as immatures on *Urtica*. Representing these, *T. urticae* is the type species of this genus. It is widely distributed throughout the Palaearctic region and is one of the most common psyllid species in Europe, feeding on several *Urtica* species, including the stinging nettle *Urtica dioica* [6,7]. Across Europe, *T. urticae* has been reported to harbour a haplotype (U) of ‘*Candidatus* Liberibacter solanacearum’, a major plant pathogenic bacterium affecting crops such as carrots and potatoes—although this haplotype U is currently thought to infect wild *Urtica* only and is considered to have little impact on crops [8,9,10,11,12,13]. *Trioza urticae* is listed as occurring in Japan in many widely referenced global databases for Psylloidea [6], but this is erroneous, because the source literature clearly states that it is not known from Japan [14,15]. In the East Asian region surrounding Japan, *T. urticae* is known from China and the Russian Far East [3,16,17]; however, there are no reliable records of this species or other nettle-feeding species based on specimen data from Japan.

In recent years, a population of *Trioza* feeding on *Urtica platyphylla* was discovered in Hokkaido, northern Japan. Detailed morphological and DNA barcoding analyses revealed that this is a new species, *Trioza miyatakei* sp. nov., that is closely related to *T. urticae* and *T. albifrons*. This is the first accurate record of a psyllid species associated with *Urtica* in Japan.

## 2. Materials and Methods

### 2.1. Taxonomy and Morphological Study

More than 70 individual psyllids were collected by sweeping and direct searches of plants in eastern Hokkaido, northern Japan, in July 2014 and June 2015. For morphological studies, most adult specimens were dry-mounted and examined under a Nikon SMZ1000 stereomicroscope (Nikon, Tokyo, Japan). Some adult specimens were slide-mounted in Canada balsam using the method described by Inoue [18] for more detailed morphological observations under a Nikon ECLIPSE E200 phase-contrast microscope (Nikon, Tokyo, Japan). All illustrations were drawn from slide-mounted specimens. Photographs of the dry-mounted adult specimens were captured with a stereomicroscope equipped with a Nikon Z50 digital camera (Nikon, Tokyo, Japan), and the serial images were stacked in Adobe Photoshop 2026 (Adobe, San Jose, CA, USA).

The morphological terminology follows Ossiannilsson [5]. The plant nomenclature follows POWO [19]. The abbreviations used for the measurements are as follows. Adults: BL, total body length measured from the anterior margin of the head to the tip of folded wings; WL, forewing length; WW, forewing width; HW, head width; AL, antennal length; VW, vertex width; VL, vertex length; GL, genal process length; MP, male proctiger length; PL, paramere length; FP, female proctiger length. Immatures: BL, body length; BW, body width including forewing pads; AL, antennal length; WPL, forewing pad length; CRW, outer circumanal ring width. In the forewing illustration, the dashed lines represent the limits of the surface spinule areas. The holotype is deposited at the Insect Museum of the National Agriculture and Food Research Organization (NARO), Tsukuba, Japan. Other materials are preserved in the author’s collection, which is presently located at the Institute for Plant Protection, NARO, Hiroshima, Japan (HIC), and partly at the Osaka Museum of Natural History (OMNH), Osaka, Japan.

### 2.2. Molecular Analysis

Non-destructive DNA extraction, PCR amplification of the mitochondrial cytochrome *c* oxidase subunit I (COI) gene fragment, sequencing, and alignment were conducted following the approaches described by Inoue [20] using the PCR primers CACF and CACR [21]. The sample information and accession numbers for the DNA sequences determined in this study, submitted to GenBank, are listed in Table 1. Pairwise distances were calculated using the Kimura 2-parameter (K2P) model [22] in MEGA12 software [23]. The maximum likelihood (ML) tree, including the sequences of *T. urticae* from England, UK (NC038113.1) and *T. albifrons* from Washington, D.C., USA (PP210906.1) obtained from the GenBank database, was reconstructed in MEGA 12 using the General Time Reversible model with 1000 bootstrap replications. The phylogenetic tree was rooted using *Trioza nigra* Kuwayama, 1910 and *Stenopsylla nigricornis* Kuwayama, 1910 as outgroups.

## 3. Results and Discussion

### 3.1. Taxonomy

#### 3.1.1. *Trioza* Foerster, 1848: 82 [24]

Type species: *Chermes urticae* Linné, 1758, by subsequent designation of Oshanin, 1912: 128 [25].

*Comments*. *Trioza* is a large genus comprising over 400 species; there are issues regarding its definition, and its polyphyletic nature has been suggested based on both morphological and molecular evidence [1,26,27,28]. There are seven species of *Trioza* associated with *Urtica* worldwide, including the new species described in this paper (Table 2); however, the true host plants [2] have not been confirmed for more than half of these based on the presence of immatures, and they are distinctly not monophyletic considering the characteristics of their wing venation and male terminalia. But the three species—*T. urticae*, *T. albifrons*, and *T. miyatakei* sp. nov.—for which *Urtica* have been confirmed as their true host are very closely related to each other, and it is reasonable to regard them as a species group. This *T. urticae* -species group shares the following combination of characteristics: antennal segments IV–X dark brown to black, forewing membrane without marginal spots, vein Rs nearly straight and almost the same length as vein M, apical half of tibiae and most tarsi dark brown to black, male proctiger unipartite, paramere nearly straight or somewhat sinuate in lateral view, apical dilation of aedeagus not hooked and smoothly rounded, female proctiger almost straight at dorsal margin in lateral view.

#### 3.1.2. Key to Adults of *Trioza* spp. Associated with *Urtica* Worldwide

1. Forewing vein Rs more than 1.2 times as long as vein M, distance between apices of veins Rs and M_1+2_ shorter than that between apices of veins M_1+2_ and M_3+4_ ………………………………………………………………………………… *Trioza huai*

- Forewing vein Rs as long as or shorter than vein M, distance between apices of veins Rs and M_1+2_ longer than that between apices of veins M_1+2_ and M_3+4_ ……………… 2

2. Aedeagus strongly hooked apically …………………………………… *Trioza frangulae*

- Aedeagus not hooked apically, rounded or somewhat quadrate apically ………… 3

3. Forewing membrane with four prominent black spots along the posterior margin ……………………………………………………………………… *Trioza quadripunctata*

- Forewing membrane without marginal spots ………………………………………… 4

4. Paramere uniformly curved anteriorly from base towards tip; female terminalia short, proctiger convex at dorsal margin in lateral view …………… *Trioza urticicola*

- Paramere nearly straight or somewhat sinuate in lateral view; female terminalia long, proctiger almost straight at dorsal margin in lateral view …………………… 5

5. Paramere without anteriorly directed apical tooth …………………… *Trioza albifrons*

- Paramere with an anteriorly directed apical tooth …………………………………… 6

6. Paramere slender and straight throughout except for base in lateral view ……………………………………………………………………………… *Trioza urticae*

- Paramere somewhat stout and sinuate in lateral view ……… *Trioza miyatakei* sp. nov.

#### 3.1.3. *Trioza miyatakei* **sp. nov.**

**Figure 1:**
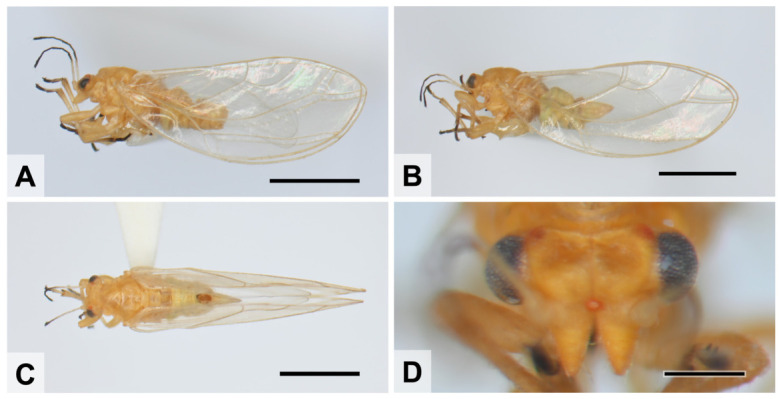
*Trioza miyatakei* sp. nov. (**A**) Male habitus, in lateral view. (**B**) Female habitus, in lateral view. (**C**) Female habitus, in dorsal view. (**D**) Head, in frontal view. Scale bars: 1 mm (**A**–**C**); 0.2 mm (**D**).

**Figure 2:**
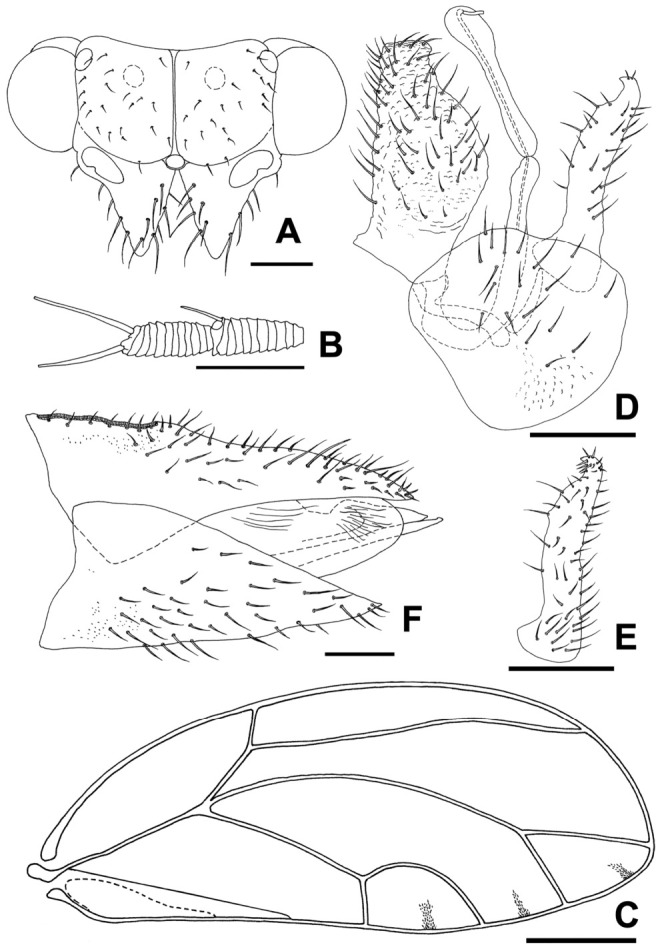
*Trioza miyatakei* sp. nov., adult. (**A**) Head, dorsal view. (**B**) Terminal two segments of flagellomere. (**C**) Right forewing. (**D**) Male terminalia, lateral view. (**E**) Paramere, inner face. (**F**) Female terminalia, lateral view. Scale bars: 0.1 mm (**A**,**B**,**D**–**F**); 0.5 mm (**C**).

**Figure 3:**
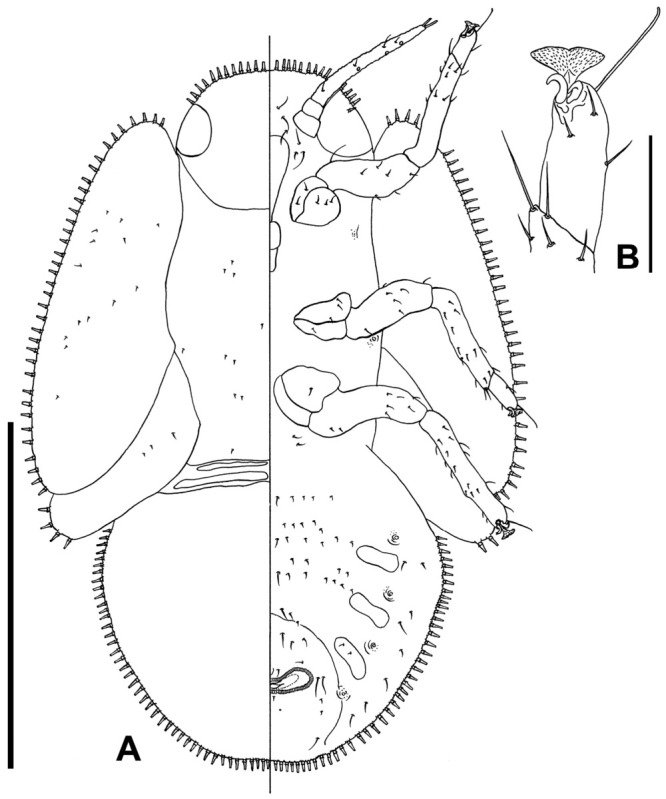
*Trioza miyatakei* sp. nov., fifth instar. (**A**) Habitus, dorsal view (left half), ventral view (right half). (**B**) Apex of tarsus. Scale bars: 0.1 mm (**B**); 1 mm (**A**).

**Figure 4:**
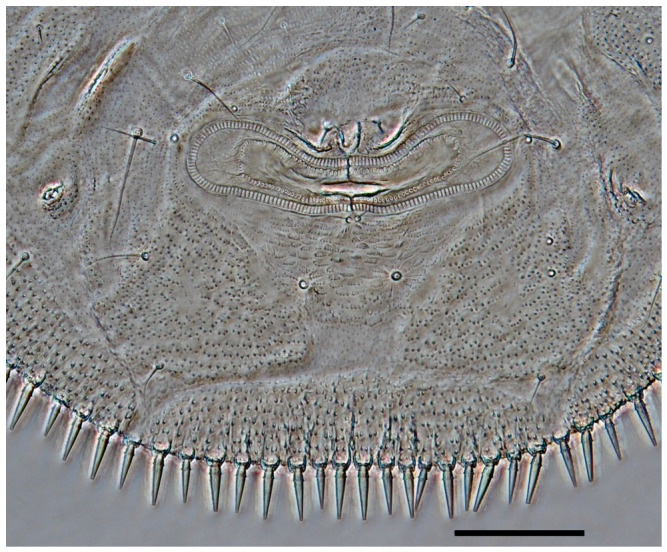
*Trioza miyatakei* sp. nov., fifth instar. Anal pore field and apex of abdomen, ventral view. Scale bar: 0.1 mm.

**Figure 5:**
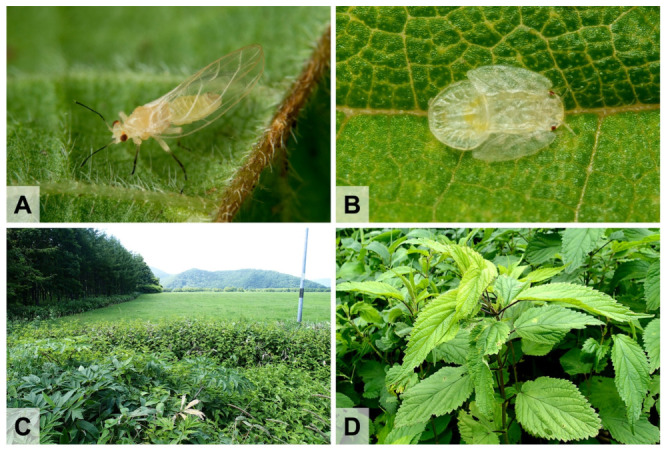
Live *Trioza miyatakei* sp. nov. and its habitat. (**A**) Adult, male. (**B**) Fifth instar immature. (**C**) Habitat (type locality), Japan, Hokkaido, Kamikawa-gun, Shimizu-chô, Shimizu, 42.992° N, 142.846° E, 280 m. (**D**) Host plant, *Urtica platyphylla*.

(Figure 1, Figure 2, Figure 3, Figure 4 and Figure 5A,B)

LSID: urn:lsid:zoobank.org:act:C016F4D4-371A-46D1-93D4-BD3324D41EF7.

Japanese name: Ezoirakusa-togarikijirami.

*Diagnosis.* Adult: Genal process (Figure 2A) almost as long as vertex (about 2/3 as long as vertex in *T. albifrons*). Antenna almost twice as long as the head width (1.5 times as long as the head width in *T. albifrons*). Paramere (Figure 2D) somewhat stout and sinuate, bent anteriorly in the middle then curved posteriorly (slender and straight throughout except for the base in lateral view in *T. urticae* and *T. albifrons*). An anteriorly directed tooth present at the apex of the paramere (lacking such teeth in *T. albifrons*). 

Fifth instar immature: Outer circumanal pore ring (Figure 3A and Figure 4), which is horizontally elongated heart-shaped and shorter in length near the body axis (uniform in length overall in *T. urticae* and *T. albifrons*). The number of marginal sectasetae (Figure 3A), which are 37-39 and 41-44 on the forewing pad and the half of the abdomen, respectively (less than 34 and 38 on the forewing pad and the half of the abdomen, respectively, in *T. urticae*).

*Additional characters. Adult.* Colouration: General colour (Figure 1 and Figure 5A) yellow to yellowish orange. Antennal segments I–III yellow, segments IV–X dark brown to black. Eye dark red. Head and thorax without distinct longitudinal stripes, at least in younger adults. Legs yellow; apical half of tibiae and most tarsi dark brown to black. Forewing colourless. Abdomen sometimes tinged light green. Apical tooth of paramere black. Mature individuals, such as overwintered adults, probably darker in general colouration; however, no such material available.

Structure: Head (Figure 2A) strongly inclined downwards, 75–80° from the longitudinal body axis in profile, slightly narrower than thorax. Vertex nearly half as long as wide; each half of the vertex strongly concave centrally and raised at the edges. Genal processes conical, 0.8–1.1 times (0.9 times in average) as long as vertex along midline, strongly divergent and acute apically. Antenna 1.9–2.1 times (1.9 times in average) as long as head width; two terminal setae of segment X nearly equal in length to each other and segment X (Figure 2B).

Forewing (Figure 2C) 2.3–2.5 times as long as wide, widest at around 2/3 from the base, bluntly acute apically; costal margin strongly arched evenly; surface spinules present only in basal area of clavus; radular spinules present in cells m_1_, m_2_, and cu_1_; areas of radular spinules often thick and rectangular in shape; vein Rs almost the same length as vein M, nearly straight, slightly sinuate in apical half; cell m_1_ 0.4 times as long as vein M; cell cu_1_ 0.5–0.6 times as long as width. Hindwing 2/3 of forewing length. Metatibia without genual spine, with four apical sclerotised spurs arranged in 3+1.

Male terminalia (Figure 2D) moderate in size. Proctiger half as long as head width, with sparse long setae; anterior margin almost straight; posterior margin weakly and roundly produced. Paramere slender, 0.8–0.9 times as long as proctiger, with sparse long setae entirely; apical tooth acute and projected cephalad; inner surface (Figure 2E) with long setae at its base and margins, with shorter retrorse setae on its central area, with three anterior-directed bristles below the apical tooth. Distal segment of aedeagus 0.7 times as long as paramere; apical dilation moderately inflated, smoothly rounded; sclerotised end tube of ductus ejaculatorius moderately long.

Female terminalia (Figure 2F) slender, moderate in length. Proctiger as long as head width, with sparse long setae, acute apically; dorsal margin almost straight, curved gently downward towards the tip; circumanal ring oval, 1/3 of proctiger length, consisting of two rows of pores of different shapes. Subgenital plate 0.9 times as long as proctiger, with sparse setae, acute apically; ventral margin slightly projected ventrad centrally.

*Fifth instar immature.* Body (Figure 3A and Figure 5B) uniformly light yellow in colour, oval in general shape, and flattened dorsally. Body marginal sectasetae narrowed towards the end and truncated at apex. Antenna three-segmented, 0.4 times as long as forewing pad; segment III (flagellum) not subdivided. Head with 13–16 pairs of sectasetae antero-marginally. Forewing pad narrow oblong-oval, 2.4 times as long as antenna; humeral lobe reaching the middle of eye. Hindwing pad with four sectasetae apically. Tarsus with claws and small, narrow fan-shaped arolium (Figure 3B). Caudal plate 0.73 times as long as wide, rounded apically. Outer circumanal ring relatively 0.25 time as wide as caudal plate, comprising a single row of elongated pores (Figure 4).

*Measurements (in mm).* Adult (5♂5♀): BL: 3.34–3.69; WL: 2.81–3.24; WW: 1.13–1.34; AL: 0.92–1.13; HW: 0.53–0.55; VW: 0.30–0.36; VL: 0.17–0.21; GL: 0.13–0.19; MP: 0.27–0.29; PL: 0.23–0.27; FP: 0.51–0.59. Fifth instar immature (one individual): BL: 2.00; BW: 1.39; AL: 0.46; WPL: 1.12; CRW: 0.24.

*Etymology.* This species is dedicated to and named in honour of Yorio Miyatake (Mr.), Japan’s most eminent psyllidologist, in commemoration of his 88th birthday.

*Host plant. Urtica platyphylla* Wedd. (Rosales: Urticaceae) (Figure 5D). The presence of immature confirms the host plant.

*Biology.* This species has only been observed as a single fifth instar immature and numerous newly emerged adults on the host plant from late June to early July, leaving many aspects of its life cycle unknown. Although closely related, *T. urticae* in the United Kingdom has up to four generations per year [29,30], the number of generations of *T. miyatakei* in Japan remains unclear. Immatures of *T. urticae* sometimes form leaf roll galls on their host plants; however, immature *T. miyatakei* have been observed to be free-living on the host plant leaf surface (Figure 5B), and no galls have been confirmed yet. The habitat of *T. miyatakei* is covered in deep snow during winter; therefore, it is thought that adults overwinter on evergreen shelter plants, especially conifers; however, no actual observations are available.

*Distribution.* Japan (Hokkaido).

*Type locality.* Japan, Hokkaido, Kamikawa-gun, Shimizu-chô, Shimizu, 42.992° N, 142.846° E, alt. 280 m (Figure 5C).

*Type material.* Holotype: ♂; JAPAN, Hokkaido, Kamikawa-gun, Shimizu-chô, Shimizu; 42.992° N, 142.846° E; 280 m; 8 Jul. 2014; H. Inoue leg.; on *Urtica platyphylla*; dry-mounted; NARO.

Paratypes: JAPAN • 21♂20♀; same data as holotype; dry- and slide-mounted, in 99.5% ethanol; HIC, OMNH • 1♂; same locality; 30 Jun. 2015; on *U. platyphylla*; H. Inoue leg.; dry-mounted; HIC • 6♂3♀; Hokkaido, Kamikawa-gun, Shimizu-chô, Haobi; 42.957° N, 142.905° E; 190 m; 30 Jun. 2015; H. Inoue leg.; on *U. platyphylla*; dry- and slide-mounted; HIC • 14♂8♀, 1 immature; Hokkaido, Nakagawa-gun, Makubetsu-chô, Chûrui-kyôei; 42.544° N, 143.289° E; 95 m; 30 Jun. 2015; H. Inoue leg.; on *U. platyphylla*; dry- and slide-mounted, in 99.5% ethanol; HIC.

*Comments. Trioza miyatakei* is very closely related to *T. urticae* and *T. albifrons*; the differences from them are noted in the diagnosis section. However, as illustrated in the literature [3,17], populations of ‘*T. urticae*’ in China and the Russian Far East possess parameres that appear to be somewhat stout and sinuously curved, potentially corresponding to *T. miyatakei* or other closely related unknown species.

To distinguish it from other *Urtica*-feeding species of *Trioza*, refer to the key in Section 3.1.2. Among the species of Triozidae associated with *Urtica*, *Triozopsis huai* Li, 2011 has been described in China [3]. The genus *Triozopsis* was proposed by Li [31]. However, because of its poorly defined and non-monophyletic nature, it was correctly synonymised with *Trioza* (*sensu lato* as in Hollis [26]) by Yang et al. [27]. However, the revised combination for *T. huai* was not mentioned in that study. Therefore, a new combination of *Triozopsis huai* with *Trioza* is proposed here as *Trioza huai* (Li, 2011) **comb. nov.**

### 3.2. Results of Molecular Analysis

**Figure 6:**
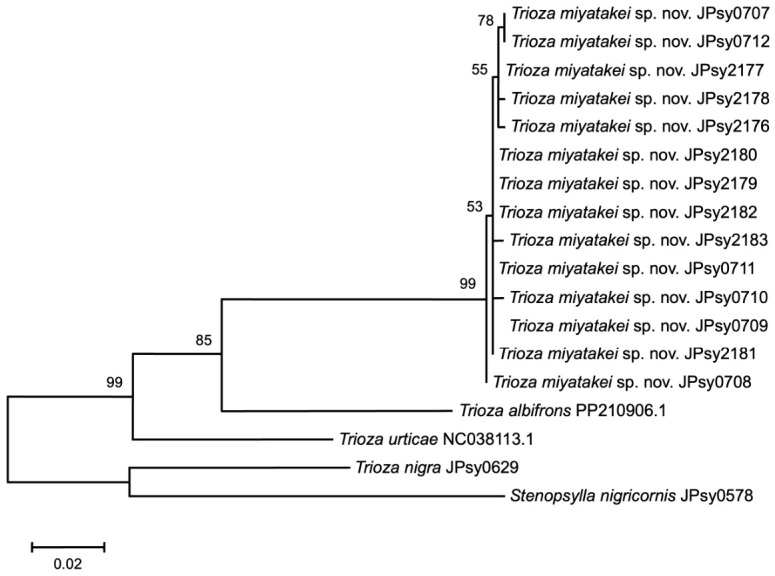
Maximum likelihood (ML) tree based on 658 bp of the mitochondrial COI sequence of *Trioza miyatakei* sp. nov. and related *Urtica*-feeders. On each branch, bootstrap support values >50% are shown. Each taxon is followed by the voucher IDs, or GenBank accession numbers for *T. albifrons* and *T. urticae*. *Trioza nigra* and *Stenopsylla nigricornis* were used as outgroups. The scale bar indicates a Kimura 2-parameter genetic distance of 0.02.

Fourteen COI sequences of 714 bp were obtained from *T. miyatakei* from three localities in Hokkaido, northern Japan (Table 1). Among *Trioza* species associated with *Urtica* worldwide, one sequence of *T. urticae* from England, UK (accession number NC038113.1) and one of *T. albifrons* from Washington, D.C., USA (accession number PP210906.1), for which sufficiently long nucleotide sequences >600 bp upstream of the COI gene are available in the GenBank database, were utilised for comparison with *T. miyatakei*. All nucleotide sequences used in the molecular analysis were aligned and trimmed to 658 bp to match the relatively short sequences obtained from GenBank. Consequently, an ML tree was reconstructed (Figure 6). *Trioza miyatakei* was supported by a 99% bootstrap value, and with the three localities being no more than approximately 60 km apart, the intraspecific genetic divergence was 0.29% on average and 0.61% at maximum (*p*- and K2P distances). Considering that an uncorrected *p*-distance of 3% is the species-separating threshold for psyllids [7,32], these three *Urtica*-feeding species exhibiting mutual interspecific divergence of ≥12% were sufficiently differentiated (Table 3). Among the three *Urtica*-feeding species, *T. miyatakei* and *T. albifrons* are sister taxa, supported by a relatively high bootstrap value of 85%; however, because only a single sequence was available for *T. albifrons*, this relationship requires reconsideration with a larger sample size.

## 4. Conclusions

*Trioza miyatakei* is likely a replacement species in Japan for the Palaearctic *T. urticae* and Nearctic *T. albifrons*. Adults of these species are morphologically similar and can be reliably distinguished only by male terminalia (especially parameres). Among these, *T. miyatakei* and *T. urticae* share greater morphological similarities, and from a geographical perspective, it is likely that the former evolved in geographical isolation from the latter. However, whilst mitochondrial COI sequences indicate that the three species are sufficiently divergent from one another, molecular phylogenetic analysis suggests that *T. miyatakei* and *T. albifrons* are sister taxa. These results are only based on a single mitochondrial marker and a limited comparative dataset; broader taxon sampling and additional genetic data would strengthen the interpretation of the phylogenetic relationships within this group.

Although *T. urticae* has been reported in some databases and checklists as occurring in Japan [6,33,34,35], these reports were not based on actual examination of Japanese materials. The examination of the insect collections preserved at OMNH, Hokkaido University, and Kyushu University, which contain major historical and comprehensive collections of Japanese psyllids, revealed no *T. urticae* specimens. Whilst it cannot be entirely ruled out that there may be undiscovered psyllid species (including *T. urticae*) in Japan that feed on *Urtica*, in addition to *T. miyatakei*, the results of field surveys and specimen examinations to date suggest that this is unlikely. Therefore, Japan is formally excluded from the distribution area of *T. urticae*. Notably, populations referred to as ‘*T. urticae*’ in China and the Russian Far East appear to share characteristics with *T. miyatakei* based on illustrations in the literature [3,17]. Consequently, populations previously recorded as ‘*T. urticae*’ in East Asia require re-examination based on both morphological and DNA barcoding approaches.

*Trioza miyatakei* is currently found only in a narrow area of eastern Hokkaido, Japan; therefore, its intraspecific genetic divergence is not precisely known. However, its host plant, *U. platyphylla*, occurs throughout Hokkaido, as well as in central and northern Honshu in Japan, and from eastern Siberia to Kamchatka in the Russian Far East [19,36]. Thus, *T. miyatakei* may be widely distributed in these regions. It should be noted that *T. urticae* and *T. albifrons* utilise the same host plant species, *U. dioica*, whilst *T. miyatakei* does not share any common host with them; however, this is due to the fact that *U. dioica* does not occur in Japan [36] and is not thought to be related to the interspecific relationships of this group.

In a genetic survey of *T. urticae* across Europe [7], the overall genetic divergence was less than 3%, falling within the range of intraspecific variation. However, a relatively large intraspecific genetic distance of 2.3% was observed in southern Europe (from Hungary to Greece). Further research on other populations of *T. miyatakei* may reveal genetic diversity comparable to that of related species.

The following new nomenclatorial acts are proposed in this study: *Trioza miyatakei* **sp. nov.**; *Trioza huai* (Li, 2011) **comb. nov.**, from *Triozopsis*.

## Figures and Tables

**Table 1:** The sample collection information and accession numbers for the COI sequences determined in this study.

Species	Plant, Collected on	Locality (Japan)	Latitude (°N)	Longitude (°E)	Date	Voucher ID	Accession No.
*Trioza miyatakei* sp. nov.	*Urtica platyphylla*	Hokkaido, Shimizu-chô, Shimizu	42.992	142.846	8 July 2014	JPsy0707	LC921148
						JPsy0708	LC921149
						JPsy0709	LC921150
						JPsy0710	LC921151
						JPsy0711	LC921152
						JPsy0712	LC921153
		Hokkaido, Shimizu-chô, Haobi	42.957	142.905	30 June 2015	JPsy2176	LC921154
						JPsy2177	LC921155
						JPsy2178	LC921156
		Hokkaido, Makubetsu-chô, Chûrui-kyôei	42.544	143.289	30 June 2015	JPsy2179	LC921157
						JPsy2180	LC921158
						JPsy2181	LC921159
						JPsy2182	LC921160
						JPsy2183	LC921161
*Trioza nigra* Kuwayama, 1910	*Stylax japonicus*	Kyushu, Fukuoka, Mt. Tachibana	33.679	130.463	1 May 2001	JPsy0629	LC921162
*Stenopsylla nigricornis* Kuwayama, 1910	*Symplocos* *nakaharae*	Kyushu, Nagasaki, Unzen	32.740	130.262	31 May 2014	JPsy1675	LC921163

**Table 2:** List of *Trioza* spp. associated with *Urtica* worldwide.

Species	Distribution	Host plant
*Trioza albifrons* Crawford, 1910	North America (USA, Canada, and Mexico)	*U. dioica*
*Trioza frangulae* Li, 2011	China (Tibet, Yunnan)	*U. trianguralis* *
*Trioza huai* (Li, 2011)	China (Henan)	*U. cannabina* *
*Trioza miyatakei* sp. nov.	Japan (Hokkaido)	*U. platyphylla*
*Trioza quadripunctata* Crawford, 1910	North America (USA and Canada)	*U. dioica* *
*Trioza urticae* (Linné, 1758)	Palaearctic (Europe, North Africa, the Middle East, and Asia excluding Japan)	*U. dioica* and several other *Urtica* spp.
*Trioza urticicola* Li, 2011	China (Shanxi)	*U. cannabina* *

* Immatures not confirmed.

**Table 3:** Mean genetic distances (%) between *Urtica*-feeding *Trioza* spp. (K2P pairwise; in parenthesis, uncorrected *p*-distance). *N* = number of samples.

	Species	*N*	1	2
1	*Trioza miyatakei* sp. nov.	14		
2	*Trioza albifrons*	1	13.25 (12.08)	
3	*Trioza urticae*	1	14.34 (12.97)	12.65 (11.60)

## Data Availability

The original contributions of this study are included in this article. Further enquiries can be directed to the author.
